# Epidemiological and genetic characterization of pH1N1 and H3N2 influenza viruses circulated in MENA region during 2009–2017

**DOI:** 10.1186/s12879-019-3930-6

**Published:** 2019-04-11

**Authors:** Hebah A. Al Khatib, Asmaa A. Al Thani, Imed Gallouzi, Hadi M. Yassine

**Affiliations:** 1 Life Science division, College of Science and Engineering, Hamad Ben Khalifah University, Doha, 34110 Qatar; 2 Biomedical Research Center, Qatar University, Doha, 2713 Qatar; 3 Biochemistry Department and Goodman Cancer Center, 3655 Promenade Sir William Osler, McGill University, Montreal, Quebec, H3G1Y6 Canada

**Keywords:** Influenza epidemics, pH1N1, H3N2, Phylogeny, Hemagglutinin, Neuraminidase, Molecular evolution

## Abstract

**Background:**

Influenza surveillance is necessary for detection of emerging variants of epidemiologic and clinical significance. This study describes the epidemiology of influenza types A and B, and molecular characteristics of surface glycoproteins (hemagglutinin [HA] and neuraminidase [NA]) of influenza A subtypes: pH1N1 and H3N2 circulated in Arabian Gulf, Levant and North Africa regions during 2009–2017.

**Methods:**

Analysis of phylogenetics and evolution of HA and NA genes was done using full HA and NA sequences (*n* = 1229) downloaded from Influenza Research Database (IRD).

**Results:**

In total, 130,354 influenza positive cases were reported to WHO during study period. Of these, 50.8% were pH1N1 positive, 15.9% were H3N2 positives and 17.2% were influenza B positive. With few exceptions, all three regions were showing the typical seasonal influenza peak similar to that reported in Northern hemisphere (December–March). However, influenza activity started earlier (October) in both Gulf and North Africa while commenced later during November in Levant countries. The molecular analysis of the HA genes (influenza A subtypes) revealed similar mutations to those reported worldwide. Generally, amino acid substitutions were most frequently found in head domain in H1N1 pandemic viruses, while localized mainly in the stem region in H3N2 viruses. Expectedly, seasons with high pH1N1 influenza activity was associated with a relatively higher number of substitutions in the head domain of the HA in pH1N1 subtype. Furthermore, nucleotide variations were lower at the antigenic sites of pH1N1 viruses compared to H3N2 viruses, which experienced higher variability at the antigenic sites, reflecting the increased immunological pressure because of longer circulation and continuous vaccine changes. Analysis of NA gene of pH1N1 viruses revealed sporadic detections of oseltamivir-resistance mutation, H275Y, in 4% of reported sequences, however, none of NAI resistance mutations were found in the NA of H3N2 viruses.

**Conclusions:**

Molecular characterization of H1N1 and H3N2 viruses over 9 years revealed significant differences with regard to position and function of characterized substitutions. While pH1N1 virus substitutions were mainly found in HA head domain, H3N2 virus substitutions were mostly found in HA stem domain. Additionally, more fixed substitutions were encountered in H3N2 virus compared to larger number of non-fixed substitutions in pH1N1.

**Electronic supplementary material:**

The online version of this article (10.1186/s12879-019-3930-6) contains supplementary material, which is available to authorized users.

## Background

Recently published data indicates that up to 650,000 deaths are reported annually due to respiratory illness associated with seasonal influenza [1]. Influenza viruses evolve rapidly, escaping natural or vaccine-induced immune response by accumulating mutations especially within surface glycoproteins genes: hemagglutinin (HA) and neuraminidase (NA) [2]. This antigenic drift results in annual influenza epidemics in humans, a major cause of morbidity and mortality and consequently increased burden on health services [3]. In addition to antigenic drift, influenza viruses evolve by genetic reassortment, leading in some occasions to life threatening pandemics. Three influenza A pandemics were reported in last century: 1918 H1N1, 1957 H2N2 and 1968 H3N2 [4, 5]. In June 2009, WHO announced the first pandemic in the twenty-first century, which was caused by swine-origin H1N1 (pH1N1) and resulted in more than 250,000 deaths worldwide [6, 7]. However, the pH1N1 virus replaced the seasonal H1N1 virus, and is currently co-circulating with H3N2 and influenza B viruses.

Antigenic drift mutations are typically localized at the antigenic sites surrounding the receptor binding site (RBS) of the HA protein. Therefore, in addition to contributing to HA antigenicity, they may affect receptor preference and virulence [8]. Four antigenic sites (Ca, Cb, Sa, Sb) have been identified for H1N1 viruses compared to five sites (A-E) for H3N2 viruses [9, 10]. Changes in HA and NA proteins may sometimes lead to acquisition of glycosylation sites, which is believed to efficiently generate antigenic variants and play critical role in virus evolution [11, 12] by masking epitopes, stabilizing HA structures and altering receptor binding specificity [13].

Each season, the World Health Organization (WHO) select virus strains for optimal vaccine performance with the recommendation for the northern hemisphere announced in February and that for the southern hemisphere in September [14]. Following 2009 pandemic, unprecedented surveillance programs have been launched to monitor influenza viruses at the molecular and epidemiological levels [15, 16].

Countries in the Middle East and North Africa (MENA) region are continuously considered as one of the hotspot regions for emerging and reemerging infectious diseases [17]. A major reason is the location of MENA countries in the pathway of three major migratory bird flyways: east Africa–west Asia, Black Sea–Mediterranean, and east Atlantic [18]. Additional risk factors that contribute in the emergence and rapid spread of epidemic diseases in the MENA region include prolonged humanitarian crises which subsequently result in massive population mobility, weak surveillance systems and limited laboratory diagnostic capacity. Further, some countries in this region, such as the Gulf States, are hubs for expatriates that constitute more than half of its population, many of which arrive from East and South-East Asia [19]. Globally shared factors also include climate change and increased human–animal interaction [20].

Until recently, little was known about epidemiology of influenza in MENA region. However, many countries have developed a systemic influenza surveillance programs after 2009 pandemic [21]. Currently, fourteen countries in MENA region are reporting to WHO virological surveillance database ‘FluNet; on weekly basis. However, there is a relatively limited number of genetic sequences available for influenza virus from MENA countries. Moreover, most of publications characterizing the molecular evolution of influenza virus in MENA originated from very few countries, mainly Egypt and Iran [22] . In the current study, we report on the epidemiology of co-circulating influenza A and B viruses in MENA since April 2009 until December 2017. Most of countries in this region are characterized by a subtropical climate [23]. However, climatic parameters such as temperature and humidity are variable among different regions of MENA and hence could affect influenza seasonality patterns. Therefore, we evaluated the epidemiology of influenza viruses among the three areas of MENA region: Gulf States, Levant, and North Africa. Additionally, we analyzed the genetic characteristics and molecular evolution of HA and NA genes of circulating pH1N1 and H3N2influenza A subtypes.

## Methods

### Data collection

For this study, we have used virological surveillance data available on WHO FluNet database (http://www.who.int/flunet) for investigating temporal and geographical distribution of influenza types A and B in MENA countries [24]. All cases reported from sixteen counties since April 2009 to December 2017 (total of 563,087 cases) were included in this study, however, no data was available from Saudi Arabia, Yemen or Libya. For further analysis on molecular evolution of influenza A subtypes (pH1N1 and H3N2), we fetched all HA and NA sequences of both subtypes depicted in Influenza Research Database (IRD) (http://www.fludb.org) by MENA countries during 2009–2017. A total of 1226 sequences were analyzed: 512 H1 sequences, 239 H3 sequences, 343 N1 sequences and 132 N2 sequences were used for sequence analysis. Influenza vaccine strains and representative strains published on the WHO collaborating center website [25] were used as references. The corresponding HA and NA sequences of were obtained from NCBI and GISAID EpiFlu Database (global initiative on sharing all influenza data (http://www.gisaid.org). The accession numbers for vaccine stains of HA and NA genes used in this study are summarized in Additional file 1: Table S1. We acknowledge the authors and submitting laboratories to all databases used in this study.

### Sequence and phylogenetic analysis

For molecular evolution analysis, sequences were compiled and edited using the DNASTAR Lasergene sequence analysis software (DNASTAR Inc. version 13.0). Multiple alignments for pH1N1 and H3N2 HA and NA sequences were carried out using ClustalW Algorithm. Herein, H1 numbering was used for H1 sequence analysis and H3 numbering scheme was used for H3 analysis (excluding signal peptide). N1 and N2 numbering were also applied for N1 and N2 sequence analysis respectively [26]. Phylogenetic trees were constructed in MEGA7, using best-fitting nucleotide substitution model, maximum likelihood method with the General time reversible model (GTR) and gamma distribution [27] . The statistical significance of the tree topology was evaluated by bootstrapping with 1000 iterations. Bootstrap probabilities higher than 50 were reported at the main branches to show support values. Clustering patterns were determined using representative influenza reference strains published on the website of the WHO Collaborating Centre, London, UK (WHO- Influenza-Centre, 2009–2016) and the corresponding HA sequences were obtained from GISAID (global initiative on sharing all influenza data) and IRD (Influenza Research Database). In addition to investigating genetic drift of HA and NA genes, we looked at possible variations in glycosylation patterns of HA and NA proteins. Potential N-linked glycosylation sites were predicted using web-based NetNGlyc 1.0 Server (http://www.cbs.dtu.dk/services/NetNGlyc/) using default settings. Only threshold values of > 0·5 were considered as potential glycosylation sites. Only scores crossing the default threshold of 0.5 were considered positive for potential glycosylation sites.

### Visualization of amino acid substitutions and glycosylation sites in HA protein using 3D models of influenza a subtypes

The identified substitutions within antigenic sites were depicted in 3D structure models using CLC Genomic Workbench. The 3D crystal structures were downloaded from protein data bank. We used 3D model of A/California/04/2009 (PDB 3LZG) as a representative of pH1N1 viruses and 2005 human H3N2 virus (PDB 2YP7) as structure model of H3N2 viruses. The antigenic sites of HAs of pH1N1 and H3N2 viruses were assigned according to Igarashi et al. (2010) and Yang et al. (2011) respectively [11, 28, 29].

### Prediction of mutations effect on HA function

For further analysis of reported mutations, we used PROVEAN software tool to predict the possible effect of identified amino acid substitution on the biological function of a HA protein (http://provean.jcvi.org). This program determines the degree of amino acid conservation in comparison to other published sequences and provides a score defining the possible effect of the substitution on protein function. A default cutoff score of less than − 2.5 indicates high probability of deleterious mutations than can affect protein’s function [30].

### Estimation of evolution rate and positive selection

The Evolutionary rate of HA and NA genes was estimated using the Bayesian Markov Chain Monte Carlo (MCMC) method as implemented in the Bayesian Evolutionary Analysis Sampling Trees (BEAST) software package v1.8.4 (http://beast.bio.ed.ac.uk) [31]. Dates of virus isolation (included in sequence names) were used to calibrate the molecular clock. For nucleotide substitution model selection, we used the recommended HKY model with gamma distributed rates. A strict molecular clock model was used with a chain length of 100,000,000-250,000,000 (relative to number of sequences) logged every 5000 states. Multiple runs were performed and combined when the number of sequences analyzed exceeded 250 sequences (H1 and N1). Results were evaluated by co-efficient of variance and 95% highest posterior density (HPD) values using the tracer v1.6 program [31]. Mutations rates were estimated for both HA and NA genes of pH1N1 and H3N2 viruses. Gene- and Site-specific selection pressures for HA and NA genes were estimated as the ratio of non-synonymous (dN) to synonymous (dS) nucleotide substitutions per site per year. Gene selective pressure was estimated by Tajima test of neutrality implemented in MEGA7. Positive selection was considered when D value is found to be positive (greater than 0). The test compares the average number of nucleotide differences between pairs of sampled sequences (referred to as pairwise difference-) and the total number of polymorphic sites (segregating site- S) in the sampled DNA sequences. The difference in the expectations for these two variables (which can be positive or negative) defines the Tajima’s D test statistic. A negative Tajima’s D indicates purifying selection while positive D signifies selection. Codon selective pressure was determined using conservative single-likelihood ancestor counting (SLAC) and fixed-effects likelihood (FEL) methods to identify codon sites under positive selection in selected HA and NA gene sequences. In all cases, positively selected sites were defined when *p* value of dN/dS ratio is less than 0.05 using the substitution model selected by website. Both methods are available at the DataMonkey online version of the Hypothesis Testing Using Phylogenies (HYPhy) package (http://www.datamonkey.org) [32].

### Statistical analysis

One-Way ANOVA followed by Dunn’s multiple comparison test was performed using GraphPad in Prism version7 software to perform statistical analysis on influenza activity of the three influenza viruses among Gulf, Levant and North Africa regions.

## Results

### Temporal distribution of influenza

According to WHO, MENA region fall within West Asia and North Africa influenza transmission zones [33]. Based on this classification, countries within the same geographical group show similar influenza transmission patterns. However, the spread and geographical location of MENA countries between tropical and temperate zones may affect seasonality patterns among MENA regions [34]. More specifically, climatic factors such as humidity, temperature and rainfalls have been shown to affect the shape of influenza seasonality in temperate zone compared to a less defined seasonality pattern in tropical zone [23]. Herein, we divided MENA region into three sub-regions: Arabian Gulf, Levant and North Africa based on differences in climate and geographical location [35] .

**Fig. 1:**
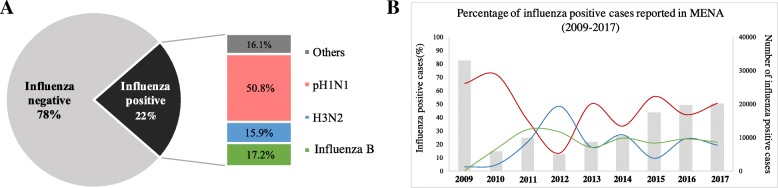
**a** Percentage of influenza types/subtypes reported to FluNet database from MENA (2009–2017). The bar chart represents the contribution of each type/subtype to the overall percentage of positive samples. **b** Overall trends of influenza activity in MENA region during 2009–2017 as reported to FluNet database. Total number of influenza positive samples received per year is presented by gray bars. Annual percentages of reported cases positive for influenza A subtypes pH1N1 (red), H3N2 (blue) and influenza B viruses are also presented

A total number of 563,087 samples were reported to WHO FluNet from sixteen countries in MENA region during January 2009 and December 2017 (Table 1). Overall, influenza positive cases accounted for 23% of all respiratory infections, with pH1N1 being the dominant virus (50.8%) followed by influenza B and H3N2 with 17.2 and 15.9% respectively (Fig. 1a). The remaining influenza cases were either not subtyped or subtyped as H5 (Fig. 1b). Unsurprisingly, the largest number of influenza positive cases were reported during 2009 H1N1 pandemic (33,064 cases) while the smallest number of positive cases were reported in 2012 (4918 cases) (Fig. 1b). During study period, pH1N1 viruses dominated in all years except in 2012 during which H3N2 viruses dominated (Table 1; Fig. 1b). After the 2009 pandemic, pH1N1 cases increased rapidly reaching up to 65.3% of influenza positive cases in 2009 thereafter peaked to highest amplitude in 2010 (72%). During 2011, however, the percentage of pH1N1 positive cases declined to 37.5%, while prevalence of H3N2 and influenza B increased gradually accounting to 30.9 and 22% of positive cases respectively (Fig. 1b). In 2013, influenza A was still the dominant type, with H3N2 being the dominant subtype representing 48.3% of the case, and only 13% were documented as pH1N1. Major activities were reported for influenza B during 2011–2012 reaching around 30%. Such activity was stable in the subsequent years. Interestingly, pH1N1 and H3N2 were showing opposite activities during following years, although pH1N1 continued to be the dominant virus (Fig. 1b).

**Table 1:** Total number of influenza A positive samples and number of HA/NA sequences analyzed in this study. Influenza surveillance data submitted from 14 countries in MENA region (2009–2017) were obtained from WHO FluNet online database. For evolution analysis, complete HA and NA sequences from pH1N1 and H3N2 viruses were downloaded from Influenza Research Database (IRD)-NCBI. Accession numbers of all vaccine strains are provided in Additional file 1: Table S1

Year	Dominant Subtype	pH1N1				H3N2			
		Number of positive samples (WHO)	Number of sequences analyzed (NCBI)		Vaccine strain	Number of positive samples (WHO)	Number of sequences analyzed (NCBI)		Vaccine strain
			H1	N1			H3	N2	
**2009**	pH1N1	21,586	132	97	A/California/07/2009	1093	22	5	A/Brisbane/10/2007
**2010**	pH1N1	4136	68	42	A/California/07/2009	280	1	1	A/Brisbane/10/2007
**2011**	pH1N1	3717	36	36	A/California/07/2009	2183	22	22	A/Perth/16/2009
**2012**	H3N2	660	27	29	A/California/07/2009	2373	37	32	A/Perth/16//2009
**2013**	pH1N1	4395	33	32	A/California/07/2009	1568	13	8	A/Victoria//361/2011
**2014**	pH1N1	3559	25	25	A/California/07/2009	2883	40	33	A/Texas/50/2012
**2015**	pH1N1	9742	157	79	A/California/07/2009	1688	47	23	A/Switzerland/9715293/2013
**2016**	pH1N1	8320	60	2	A/California/07/2009	4784	47	8	A/Hong Kong/4801/2014
**2017**	pH1N1	10,143	–	–	A/Michigan/45/2015	3891	10	–	A/Hong Kong/4801/2014
**Total**		66,228	538	342		20,743	239	132

**Fig. 2:**
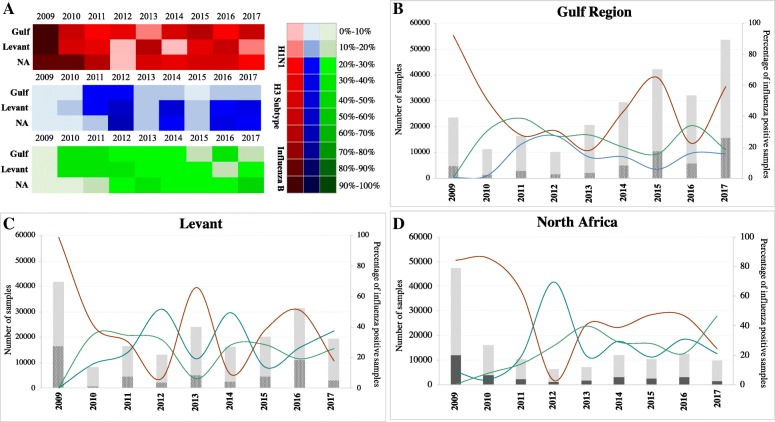
Influenza activity patterns of co-circulating influenza A subtypes (pH1N1 and H3N2) and influenza B in MENA, 2009–2017. **a** Heat map showing the levels of influenza activity of each type/subtype in each region (*n* = 3) from April 2009 to December 2017 period. The color scale denotes the percentage of influenza positive samples relative to corresponding color shade. Annual activity trends of influenza viruses: pH1N1 (red), H3N2 (blue) and influenza B in Gulf (**b**), Levant (**c**) and North Africa (**d**) regions since April 2009 to December 2017. Bars represent the total number of samples collected in each year while doted pattern in each bar denotes number of influenza positive samples in each specific year

The mean proportions of influenza positive cases reported during 2009–2017 had shown no significant differences among Gulf, Levant and North Africa regions (*P* value 0.39). More specifically, the mean proportion of pH1N1 and influenza B cases did not differ significantly (*P* value 0.85 and 0.988 respectively) among regions under the study. However, a significant difference was found in percentage of H3N2 cases (*P* value 0.0158) between Gulf and North Africa. Although the overall epidemiological trend of influenza A and B viruses were similar among the three regions, minor variations were observed after 2011 (Fig. 2). Although the activity of pH1N1 was the lowest among other viruses during 2012 in Levant and North Africa, all three viruses were reported to have similar prevalence in Gulf during 2012. During following years, variable influenza activity was seen of the three viruses among the three regions (Fig. 2a). In Levant, pH1N1 and H3N2 viruses were showing alternative activities with pH1N1 dominating in 2013, 2015 and 2016 while H3N2 dominating in 2012, 2014 and 2017 (Fig. 2b). On the other hand, pH1N1 remained the dominant virus during 2013–2017 in both Gulf and North Africa region (Fig. 2b and d). Influenza B showed similar activity trends to that of H3N2 in Gulf and Levant except in 2013 and 2015, during which, influenza B was showing similar trend to that of pH1N1. However, opposite activity patterns of the influenza B and H3N2 viruses were observed in North Africa during the same period (2013–2017) (Fig. 2c). Of note, pH1N1 positive samples dropped by half in Levant (18%) and North Africa (21%) regions in 2017 while increased to 59% in Gulf (Fig. 2b-d).

The well-established trend of influenza A was similar to that of Northern Hemisphere with a single consistent peak in all epidemics of all three regions (Additional file 2: Figure S1) [36]. The peak period, however, commenced in October in Gulf and North Africa while started later in December in Levant. During epidemics, influenza-positive cases rapidly increased from October and peaked in December–January in Gulf and North Africa. In contrast, highest influenza activity was reported during January–February in Levant. Additionally, the duration of influenza activity was similar in Gulf and North Africa; in which the activity continued for a longer period (October–April) compared to a sharper peak in Levant (December–March). Interestingly, the typical seasonal influenza peak was altered during specific epidemics. In North Africa, for example, the influenza activity peaked during February–March in 2013 and 2014 epidemics instead of the classical peak in December–January. Moreover, a late secondary peak with high amplitude (77%) was observed in Levant during February–May in 2015 following an early primary peak (amplitude of 17%) in December. Finally, a stable influenza activity was noticed during December to April of 2014 and 2015 in Gulf with no distinctive primary peak observed (Additional file 2: Figure S1).

### Molecular evolution analysis of HA and NA genes

#### Evolution rates and positive selection

The mean evolution rate of the H1 and H3 genes of pH1N1 and H3N2 viruses analyzed in this study amounted to 1.91 × 10^− 3^ and 1.47 × 10^− 3^ substitutions per site per year respectively. For NA genes, values of 1.92 × 10^− 3^ and 3.09 × 10^− 3^ substitutions per site per year were obtained for the N1 and N2 respectively (Table 2). Selective pressure acting on HA and NA genes showed that both genes of pH1N1 and H3N2 were under negative selection (*P* value of pH1N1: HA = − 2.13 and NA = − 2.19) and (P value of H3N2: HA = − 1.92 and NA = − 1.95). Besides, the positive selection at codon level was estimated by assessing the overall number of non-synonymous (dN) to synonymous substitutions (dS). Analysis showed that the overall dN/dS values of the coding HA regions of pH1N1 and H3N2 were 0.32 and 0.28, respectively (Table 2). Two sites in H1 gene were found to be under positive selection by two computational models (SLAC and FEL) with significant statistical *p*-values (< 0.05 for both). The two amino acids which were found to be prone to adaptive evolution were 239 and 240 in Ca antigenic site. These two residues are located in an overlapping position of antigenic and receptor binding site [29]. In particular, amino acid in 239 position, is a key determinant for virus binding to sialylated glycan receptors [37]. Additionally, amino acids in 202 and 468 positions corresponding to receptor binding site and stem respectively, were also found to be under positive selection using FEL method. Two codons in the signal peptide (positions 10 and 13) and three codons in stem were additionally identified to be under diversifying positive selection. On the other hand, applying FEL and SLAC methods on H3 genes resulted in different outputs regarding residues under positive selection. While 239 position was found to be under positive selection using SLAC method, position 16 and 69 were determined to be positively selected by FEL method (*p* value < 0.05). None of residues of NA genes of both subtypes were reported to be under positive pressure.

**Table 2:** Estimation of evolution rate and positive selection for HA and NA genes of pH1N1 and H3N2 viruses. Evolution rates were estimated using the Bayesian Markov Chain Monte Carlo method in the Bayesian Evolutionary Analysis Sampling Trees (BEAST) package (v1.8.4). HPD represents lower and upper limits of highest posterior density that contains 95% of the sampled values. The selective pressure at codon level was also calculated using the most conservative ‘single-likelihood ancestor counting’ algorithm (SLAC) and the ‘fixed effects likelihood’ (FEL) methods available at the DataMonkey web server of the HYPHY software. (N) refers to the number of positively selected amino acids and (AA) refers to amino acids that were found to be under positive selection. Positively selected sites were defined when dN/dS p value is less than 0.05 using the best fit model as recommended by website

	Number of analysed sequences	Nucleotide substitution rate (×10^−3^)		Positive selection					
		Mean	95% HPD	SLAC			FEL		
				Mean (dN/dS)	N	AA	Mean (dN/dS)	N	AA
H1	512	1.91	1.68–2.1	0.336	3	10–239-240	0.311	8	13–202–239-240-305-468-543-546
H3	239	1.47	1.27–1.68	0.299	1	239	0.267	2	19–69
N1	342	1.93	1.7–2	0.352	–	–	0.311	–	–
N2	132	3.09	2.5–3.1	0.238	–	–	0.208	–	–

#### Phylogenetic tree analysis

**Fig. 3:**
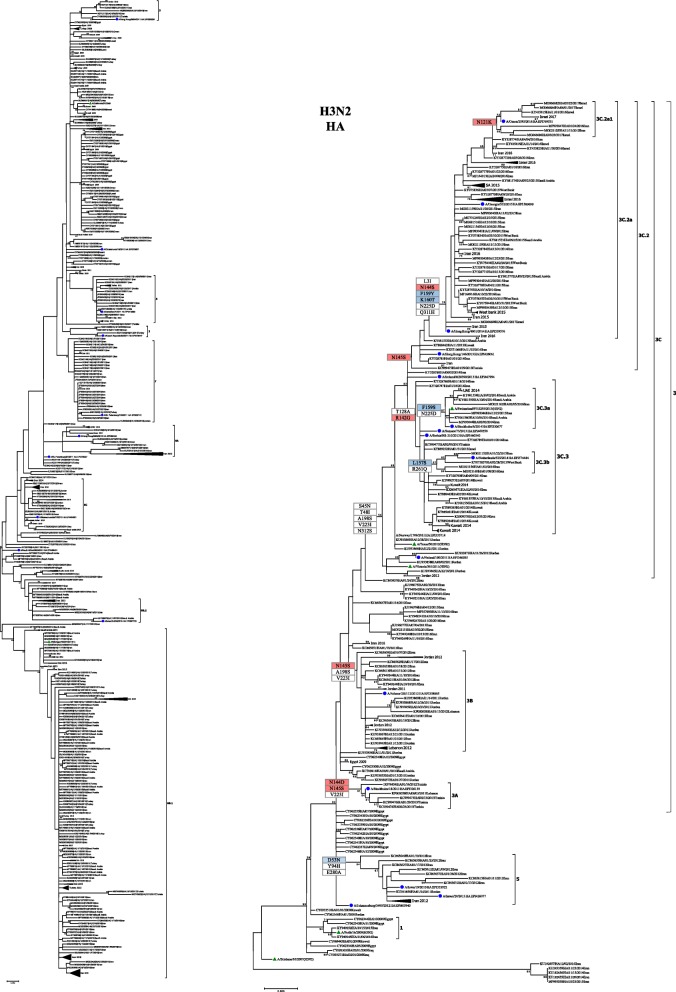
Phylogenetic trees of HA nucleotide sequences of pH1N1 and H3N2 viruses. HA sequences of pH1N1 (*n* = 512) and H3N2 (*n* = 239) reported from MENA region between 2009 and 2017 were compared to vaccine strains recommended by WHO (denoted with green triangles) and the reference strains of known clades as reported in WHO Influenza Center London, indicated by navy blue circles [25]. Phylogenic trees were generated using maximum likelihood method by GTR + G model using in MEGA7 software. Bootstrap values of 1000 replicates and only values larger than 50 are indicated at the nodes. At the major nodes are the signature amino acid changes in different colors according to antigenic site: Red for Sa (pH1N1) and A (H3N2), blue for Ca (pH1N1) and B (H3N2), green for C (H3N2). Scale bar represents approximately 0.005 nucleotide difference between close relatives

To assess the evolution pH1N1 and H3N2 viruses during the same period, HA sequences of circulating strains were used to construct phylogenetic trees along with the vaccine and reference sequences. The phylogenetic trees of HA genes of both subtypes formed phylogenetic clusters representative of each season between 2009 and 2017. The phylogenetic analysis of HA sequences revealed clustering patterns similar to those reported in the annual and interim reports of WHO since 2009 until 2016 [25]. Analysis of pH1N1 viruses revealed seven genetic groups that evolved between 2009 and 2017 (Fig. 3). In 2009, the majority of viruses belonged to the HA clade 1 represented by the vaccine strain A/California/07/2009, however, minority of viruses (7.6%) were also found to belong to clade 4. In contrast, pH1N1 viruses isolated during 2010 were showing more diversity by clustering within four genetic clades: clade 1 (37%), clade 3 (7%), clade 5 (10%) and clade 7 (26%). In 2011, viruses within clades 5 and 7 were predominant (50 and 28% respectively) in addition to clades 2, 3 and 6B which represents 27% of viruses in 2011. Following 2011, all pH1N1 viruses belonged to clade 6 and its related sub-clades (6A, 6B and 6C). Clade 6 is characterized by carrying amino acid substitutions: D114N, S202 T, S220 T and K300E in HA1 and E391K, S468 N and E516K in HA2. In 2012, pH1N1 viruses were mainly found within subclades 6A (44%) and 6C (26%). Viruses belonging to both clades continued to circulate during 2013, however, minority of viruses were also found to belong to sub-clade 6B which has the additional substitutions: K180Q and A273T in HA1 and E516K in HA2. The HA sequences of pH1N1 viruses isolated in 2014 clustered mainly within clade 6B (80%) and sub-clade 6B.2 (12%) which is characterized by E508G substitution in HA2. In 2015, clade 6B.1 became the predominant genetic group, while only 5% of viruses belonged to 6B.2 sub-clade. Viruses within 6B.1 group characterized by S101 N, S179 N and I233T in HA1 as compared to parental clade 6B. All Viruses collected in 2016 and 2017 (only 3 sequences found in IRD) fell into genetic clade 6B.1.

On the other hand, phylogenetic analysis of H3 gene clustered viruses into three genetic groups in which vaccine strain A/Perth/16/2009 represent clade 1 (Fig. 3). H3N2 viruses in 2009 clustered with A/Perth/16/2009, the vaccine strain recommended during 2010–2012. During 2011 and 2012, H3N2 strains belonged to sub-clades of genetic group 3 (3A, 3B and 3C) which share amino acid substitutions: N145S, V223I in HA1 and D487N in HA2. Of the genetic group B, H3N2 viruses belonging to sub-clade 3B dominated in 2011 (41%) and in 2012 (54%). Moreover, 32% of sequences in 2012 fell into clade 5 which is characterized by D53N, Y94H and E280A. H3N2 strains from 2013 and 2014 grouped mainly into 3C.3 and 3C.2 sub-clades. In 2013, 38% of viruses were found within 3C.3 related subclades (3C.3a and 3C.3b), 23% within 3C.2 and 23% within 3A. Similarly, majority of viruses sequenced in 2014 belonged to sub-clade 3C.3 (65%) compared to 12.5% belonged to 3C.2 sub-clade. All H3N2 viruses of 2015 and 2016 belonged to sub-clade 3C. 2 as defined by N145S substitution. Of these, more than 90% were grouped within 3C.2a sub-clade that is characterized by additional substitutions such as L3I, N144S, K160 T and N225D. By the end of 2015, a novel subgroup of viruses emerged within 3C.2a subclade, 3C.2a1, with majority of viruses belonging to 3C.2a1 subclades carrying N121K substitution. All H3N2 viruses isolated from MENA region in 2017 were found to belong to 3C.2a1 subclade.

### Amino acid sequence variation in HA and NA proteins

Here, amino acid sequences for HA and NA proteins of pH1N1 and H3N2 viruses circulated in MENA region during 2009–2017 were compared to vaccine strains of respective years. Genetic characterization was performed by sequencing analysis of 512 HA and 343 NA sequences of pH1N1; and 239 HA and 132 NA sequences of H3N2 viruses. Full length HA and NA proteins were used for alignment and subsequent analysis: the length of HA and NA genes were 566 and 469 amino acids for both viruses respectively.

### Genetic characterization of pH1N1 viruses

#### Molecular evolution of H1 protein

Alignment of 512 HA amino acid sequences of pH1N1 viruses revealed multiple amino acid changes in comparison to vaccine strain A/California/07/2009. Overall, amino acid sequences of full HA protein were highly conserved showing > 98% similarity to Cal09 virus in all pH1N1 viruses isolated since pandemic in 2009 until 2017. Comparison of HA antigenic sites of 2016 pH1N1 with vaccine strain is shown in Table 3. Two substitutions at 179 and 180 positions were found in Sa antigenic site that showed 84.6% similarity to Cal09 vaccine virus. Sb and Ca sites, on the other hand, showed 91.7 and 94.7% similarities to A/California/07/2009 virus respectively during 2016 while no substitutions were detected in Cb site (Table 3).

**Table 3:** Identification of amino acid substitutions in H1 antigenic sites of pH1N1 viruses. Antigenic sites of HA sequences were compared to vaccine strains A/California/07/2009. Amino acids in *italics* indicate amino acids that differ from vaccine sequence. Bold amino acids denote amino acid changes found in vaccine strain recommended for subsequent season (A/Michigan/45/2015). A/California/07/2009 was used in influenza vaccine composition since the emergence of pandemic H1N1 in 2009 before being replaced by A/Michigan/45/2015 in 2016. Similarity of antigenic sites between vaccine strains and circulating viruses is shown as percentages in the last column

H1					Similarity to pH1N1 vaccine strain (%)		
	Sa		Sb	Ca			
	179	180	202	220			
California/2009	S	K	S	S	Sa	Sb	Ca
2016	*N*	*Q*	*T*	*T*	84.6	91.7	94.7
Michigan/2015^a^	N	Q	T	T			

^a^A/Michigan/45/2015 vaccine was recommended to be used in 2016–2017 season, however, only 3 sequences were depicted in NCBI-IRD from MENA region during 2017 therefore sequences isolated in 2016 were used for comparison purpose

In total, 26 substitutions were reported in HA protein; 15 in HA globular head and 6 in the stem region. Seven of identified substitutions were found in antigenic sites including S179 N and K180Q in Sa site; S202 T in Sb site and A203T, S220 T, R222K, D239G/E/N and Q240R in Ca site (Table 3). Substitutions were also detected sporadically near antigenic sites such as Q240R and G187E/R substitutions near the Ca site and A214T substitution near to the Sb site. Majority of amino acid residues within RBS were highly conserved showing only four substitutions: V149E and A151T in 130-loop; S202 T and A203T in the 190-helix and D239N/E/G in the 220-loop (Table 4). Additionally, two substitutions were also observed near RBS: S200P and I233T that have been identified to alter receptor binding affinity. Ten of the amino acid substitutions were successfully introduced into the viral population in 2015, and were identified in majority of viruses (> 80%) circulating in subsequent seasons. Half of these substitutions are located in HA head (S179 N, K180Q, S220 T, I233T and A273T) while the other half are located in HA stem region (D114N, K300E, E391K, S468 N and E516K).

**Table 4:**
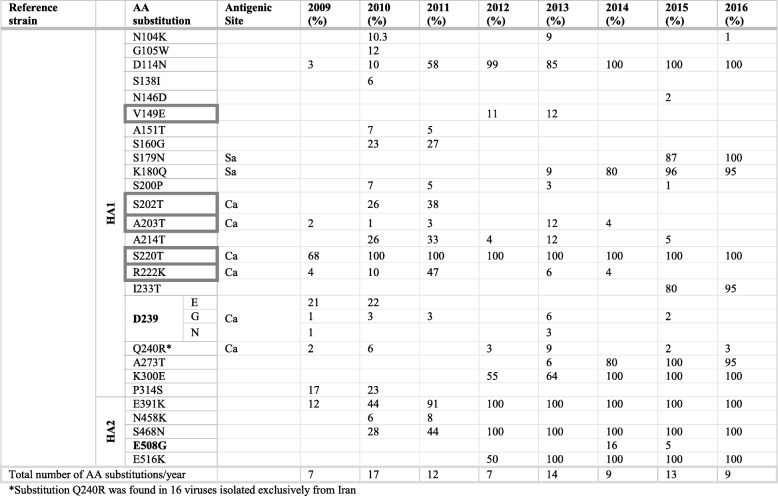
Amino acid substitutions identified in HAs of pH1N1 viruses sequenced during 2009–2017. Amino acid substitutions of pH1N1 are reported in comparison to A/California/07/2009. Bordered cells indicate substitutions in receptor binding site. Deleterious mutations were predicted by online server PROVEAN and are indicated in bold

*Substitution Q240R was found in 16 viruses isolated exclusively from Iran

**Fig. 4:**
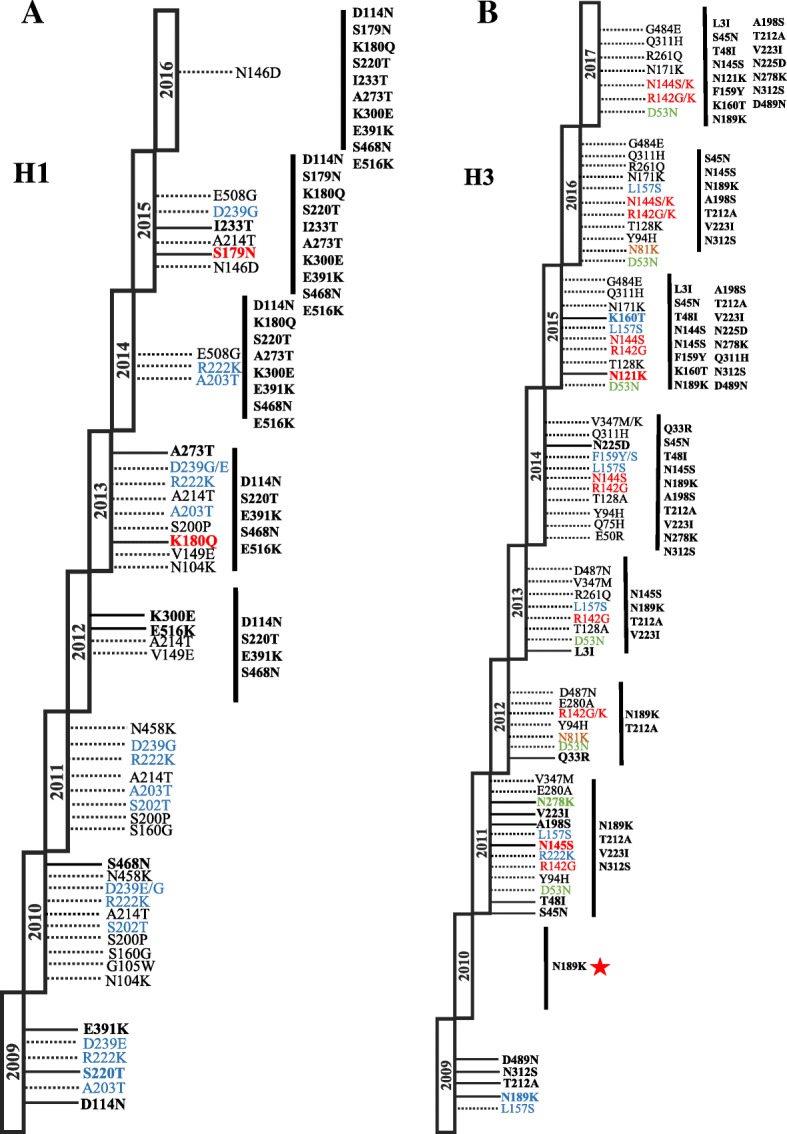
Summary of amino acid substitutions identified in HA proteins of (A) pH1N1 viruses and (B) H3N2 viruses between 2009 and 2017 in MENA. Substitutions are colored with respect to their localization in the antigenic sites. For pH1N1: Sa is colored red and Ca is colored blue. For H3N2: A is colored red; B is colored blue; C is colored green and D is colored orange. Sporadic and fixed substitutions (bold) are indicated by the dashed and continuous lines respectively. Fixed substitutions that have been found in > 80% of viruses are listed in parallel (on the right) to respective year. Only one HA sequence has been found in IRD representing H3N2 virus from MENA during 2010 (red star)

In 2009, the majority of viruses (68%) emerged with S220 T substitution at Ca site. Additional substitutions were also reported with moderate frequencies such as D239E (21%), P314S (17%) and E391K (12%) while only minority of viruses were carrying D114N (3%), A203T (2%) and R222K (4%) substitutions in respective season (Table 4). Of these substitutions, S220 T and E391K as well as D114N were the genetic signature of subsequently circulating viruses. Viruses isolated in 2010 were characterized by the emergence of S160G (23%), S202 T (26%), A214T (26%) substitutions. Additionally, a minor group of viruses had N104K (10%), S200P (7%) and N458K (6%) substitutions. The later substitution (N458K) was predicted by PROVEAN to exert deleterious effect on HA1 function (score − 4.98). Further, two of previously circulating variants, D239E (22%) and P314S (23%), were lost from viruses isolated after 2010. Two of detected substitutions: G105 W (12%) and S138I (6%) were introduced in the viral population and were only found in viruses isolated in 2010 (Fig. 4a). During 2011, no additional variants were encountered in pH1N1 viruses, instead, previously reported mutations kept circulating with variable frequencies. Forty-seven percent of the strains had R222K substitution at the Ca site and 33% had A214T substitution. S200P substitution was also observed in minority of viruses (5%). Importantly, 8 and 3% of circulating pH1N1 strains in 2011were detected with D239G and N458K mutations respectively, both of which were predicted to be have deleterious effect on HA1 by Provean. S160G, S202 T and N458K were not detected in subsequent influenza seasons in MENA region. Despite the minor pH1N1 activity during 2012, new viruses evolved carrying additional substitutions: E516K and V149A (11 and 50% respectively) in addition to K300E which was encountered in more than 50% of viruses. Along with E516K, K300E substitution in stem domain became fixed in subsequent seasons. All viruses isolated during 2012 were also carrying D114N, S220 T, E391K and S468 N substitutions. During 2013 season, all viruses were characterized by S220 T/E391K/E516K changes (Fig. 4a). Two additional substitutions, K180Q and A273T, was detected in in 9 and 6% of viruses respectively during 2013 season, however both became characteristic for the subsequent pH1N1 viruses isolated during following seasons (Fig. 4a). The deleterious mutation D239G was also detected at low frequency (6%) in addition to previously circulated mutations such as S200P (3%), A203T (12%) and A214T (12%). As 2014 year started, five substitutions were found in 100% of viruses isolated in subsequent seasons: D114N, S220 T, E391K, S468 N and E516K. Furthermore, 16% of viruses in this season had E508G substitution in the HA2 subunit (Table 4). In 2015, the majority of viruses were characterized by two novel variants, S179 N (87%) and I233T (87%), which gradually replaced original amino acids and became dominant variants in 2016. Viruses circulated during 2016 season were more stable compared to previous seasons with no novel variants detected. Instead, almost all viruses isolated in this season (95–100%) were carrying fixed mutations that has been established in previous seasons such as D114N, S179 N, K180Q and I233T in HA1 subunit and E391K, N468S and E516K in HA2 subunit (Fig. 4a).

**Fig. 5:**
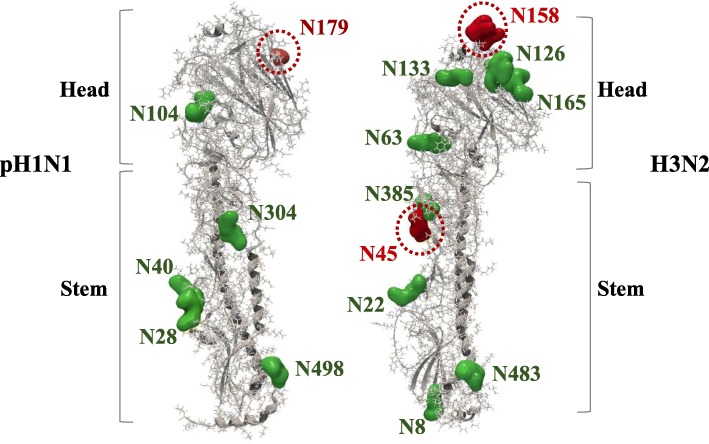
The glycosylation sites of HA proteins of pH1N1 and H3N2 viruses. The 3D structures of HA monomers from pH1N1 (PDB 3LZG) and H3N2 (PDB 2YP7) are displayed using CLC genomic workbench version 11. The 3D structures show glycosylation sites with respect to their localization in the HA protein: conserved glycosylation sequon are colored green and acquired glycosylation sequon are colored in red

Glycosylations of HA protein can also alter antigenic properties of the virus by masking antigenic sites and preventing antibodies recognition [38]. Seven potential glycosylation sites were found in the HA protein of pH1N1 strains, including N28, N40, N104, N304, N498 and N557 which are conserved in all H1 sequences (Table 5) [39]. Interestingly, the appearance of N179 residue at Sa site in 2015 has led to acquisition of a novel potential glycosylation site at head of HA protein (Fig. 5). Potential glycosylation at N179 site located in HA globular head was first detected in 2% of HA sequences during 2011. However, 85 and 99% of HA sequences have acquired N179 glycosylation sequon during 2015 and 2016, respectively (Table 5). An additional glycosylation site, N489, was detected in sequences isolated from Iran during 2013 (18%) and 2016 (2%) (Table 5). Of note, glycosylation sequons at N28, N104, N498 and N557 were lost from viruses isolate from Iran at different time periods. In 2015, for example, N28 and N557 sequons were missing from 1.5 and 5% of HA sequences, respectively. N104 glycosylation was also missing from 2% of HA sequences, all of which have been isolated from Iran during 2010, 2013 and 2016.

**Table 5:** Dynamics of HA and NA glycosylation for pH1N1 viruses isolated from MENA region during 2009–2017. In total, glycosylation patterns of 512 HA and 343 NA sequences of pH1N1 virus were analyzed. Numbers between brackets represent the percentage of sequences expressing glycosylation sequon in corresponding year while conserved glycosylation sites are indicated in bold

Localization of potential glycosylation sequons in HA and NA of pH1N1 viruses												
HA												
		Amino acid position										
Year	Number of sequences analyzed	Stem		Head			Stem					
2009	132	**28**	**40**	**104**			**304**			**498**	**557**	
2010	68	**28**	**40**	**104**			**304**			**498**	**557**	
2011	36	**28**	**40**	**104**		179 (2%)	**304**			**498**	**557**	
2012	27	**28**	**40**	**104**			**304**			**498**	**557**	
2013	33	**28**	**40**	**104**			**304**		489* (18%)	**498**	**557**	
2014	25	**28**	**40**	**104**			**304**			**498**	**557**	
2015	157	**28**	**40**	**104**		179 (85%)	**304**			**498**	**557**	
2016	60	**28**	**40**	**104**		179 (99%)	**304**		489* (2%)	**498**	**557**	
NA												
Year	Number of sequences analyzed	Amino acid position										
2009	97			44 (11%)	**50**	**58**	**63**	**68**	**88**	**146**	**235**	**386**
2010	42		42 (36%)		**50**	**58**	**63**	**68**	**88**	**146**	**235**	**386**
2011	36		42 (100%)		**50**	**58**	**63**	**68**	**88**	**146**	**235**	386*
2012	29	28 (7%)	42 (100%)		**50**	**58**	**63**	**68**	**88**	**146**	**235**	**386**
2013	32		42 (100%)		**50**	**58**	**63**	**68**	**88**	**146**	**235**	**386**
2014	25		42 (100%)		**50**	**58**	**63**	**68**	**88**	**146**	**235**	**386**
2015	79		42 (100%)		**50**	**58**	**63**	**68**	**88**	**146**	**235**	**386**
2016	3		42 (100%)		**50**	**58**	**63**	**68**	**88**	**146**	**235**	

HA Glycosylation: glycosylation site, N489, was detected in sequences isolated from Iran. Glycosylation sequons at N28, N104, N498 and N557 were lost from viruses isolate from Iran at different time periods. In 2015, for example, N28 and N557 sequons were missing from 1.5 and 5% of HA sequences, respectively. NA Glycosylation: N386 glycosylation sequon was missing from one sequence in 2011, however, during following years more NA sequences were found to lack 386 glycosylation (31% of sequences in 2012; 80% in 2014; 77% in 2015) before disappearing completely from NA sequences in 2016

#### Molecular evolution of N1 protein

A total of 343 NA protein sequences of pH1N1 viruses were analyzed in comparison to NA of vaccine strain Cal09 (KC781784). The catalytic sites represented by the following residues: 118R, 119E, 151D, 152R, 179 W, 223I, 225R, 277E, 293R, 368R, and 402Y as well as framework residues which support the catalytic residues: 156R, 180S, 228E, 247S, 278E, and 295 N [40, 41] were highly conserved in all pH1N1 viruses. However, I117M (3%), N270K (10%) and N369K (47%) substitutions were found near the catalytic sites, whereas N248D (93%) and N270K (10%) substitutions were encountered close to the framework sites (Additional file 3: Table S2). Fourteen distinct substitutions were observed at NA antigenic sites denoted by NA residues 83–143, 156–190, 252–303, 330, 332, 340–345, 368, 370, 387–395, 400, 431–435, and 448–468 [42]. Most common substitutions in antigenic sites were V106I (57.5%) followed by K432E (23.6%). The latter was detected in majority pH1N1 viruses in 2015. Additional substitutions in antigenic sites were N68 T (5%); H275Y (4%); N341D (3%) and E462K (6%) (Summarized in Additional file 3: Table S2). In addition to K432E substitutions mentioned earlier, five of emerged substitutions predominated in 2015: N200S, V241I, N248D, I314M, N386K and N369K. Of these, V241I, N369K, N386K and K432E, alone or in conjunction with oseltamivir resistance variant, H275Y, have been reported to significantly affect binding affinity of oseltamivir to neuraminidase, rendering neuraminidase less susceptible [43, 44].

Interestingly, the presence of I46T mutation in 11% of NA sequences in 2009 resulted in the acquisition of potential glycosylation at N44 residue. In total, eight glycosylation sites were found in N1: N50, N58, N63, N68, N88, N146, N235 and N386. Of these, N68 glycosylation was lost form 16% of pH1N1 viruses isolated from Lebanon during 2009. Similarly, N368 glycosylation sequon was missing from one sequence in 2011, however, during following years more NA sequences were found to lack 386 glycosylation (31% of sequences in 2012; 80% in 2014; 77% in 2015). The potential N-linked glycosylation sites in NA of pH1N1 viruses (2009–2017) are shown in Table 5.

### Genetic characterization of H3N2 viruses

#### Molecular evolution of H3 protein

In order to track the molecular evolution of H3N2 viruses circulated in MENA region during 2009–2017, the potential genetic drift in HA and NA genes were investigated. Complete 239 HA protein sequences were analyzed and compared against vaccine strains of the respective years. The overall HA amino acid identities among the H3N2 strains isolated since 2009 compared to the respective vaccine strains were > 98%. The similarities of analyzed HA sequences were estimated to range from 99.1% in 2009 to 98.6% in 2017. Antigenic epitope A, was the most variable compared to vaccine strains reaching 77% identity to 2017 vaccine strain Hong Kong/2014, with A3 site being the least conserved compared to A1 and A2 sites (Table 6). B antigenic epitope had 89.5% similarity to vaccine strains in all years except for viruses circulated in 2012 which had 100% similarity to Perth/2009 vaccine strain. Antigenic epitope C of 2016 viruses was only 66.6% conserved compared to vaccine strain (Switzerland/2013) while it had 100% similarities to vaccine strains in other years (2010–2015-2017). Similarities in D epitope ranged from 88% in 2012 with two substitutions, to 100% in subsequent years. The most conserved epitope of all was the E site, with no substitutions detected throughout the study period. Of interest, variants at two antigenic sites changed over time. Specifically, amino acid residue 144 of the antigenic epitope A3 which was asparagine in 2009 strains, was then mutated to Serine in all viruses isolated in 2015 and then to either Lysine (30%) and Serine (60%) of sequences isolated in 2017. A similar variation pattern was observed for amino acid 159 of B1 epitope. This amino acid residue was phenylalanine between 2009 and 2013, and then mutated to Tyrosine or Serine in 30% of HA sequences in 2014, and finally to Tyrosine in 2017 (Table 6).

**Table 6:** Identification of amino acid substitutions in H3 antigenic sites of H3N2 viruses. Antigenic sites of H3 protein were compared to vaccine strains Brisbane/2007**.** Here, we are presenting changes in antigenic sites of viruses isolated following any modification in H3N2 vaccine strain (2012, 2014, 2015, 2016 and 2017). Dash (−) indicates amino acid similar to vaccine sequence while amino acids different than vaccine strain are indicated in *italics*. Bold amino acids denote amino acid changes found in vaccine strains recommended for subsequent season in comparison to Brisbane/2007. Similarity of antigenic sites between vaccine strain and circulating viruses is shown as percentages in the last column

H3																												Similarity to vaccine strains (%)				
	A1		A2	A3						B1								B2		C1		C2		E1	D							
	121		135	142		144		145		157		158		159			160	189	194	53		278		62	212		215					
Brisbane/2007	N		T	R		N		N		L		K		F			K	N	L	D		N		E	T		P	**A**	**B**	**C**	**E**	**D**
2009	–		–	–		–		–		–		K	*N*	–			–	*K*	–	–		–		–	–	*A*	–	100	89.5	100	100	97
Perth/2009	N		T	R		**K**		N		L		**N**		F			K	**K**	L	D		N		**K**	T		**S**					
2012	–		–	–		K	*N*	N	*S*	–		–		–			–	–	–	D	*A*	*P*		–	*A*		*P*	*90*	*100*	*84*	100	*88*
Victoria/2011	N		T	R		N		N		L		**N**		F			K	**K**	L	D		N		E	**A**		P					
2014	–		–	*G*		–		*S*		L	*S*	–		F	*Y*	*S*	–	–	–	–		–		–	–		–	90	89.5	84	100	100
Texas/2012	N		T	R		N		N		L		**N**		F			K	**K**	L	D		**K**		E	**A**		P					
2015	–		–	R	*G*	*S*		*S*		–		–		*Y*			*T*	–	–	–		–		–	–		–	86.3	89.5	100	100	100
Switzerland/2013	N		T	**G**		N		**S**		L		**N**		**S**			K	**K**	L	D		**K**		E	**A**		P					
2016	N	*K*	–	G	*R*	*S*	*K*	–		–		–		*F*	*Y*		–	–	–	–	*N*	K	*N*	–	–		–	82	89.5	66.6	100	100
Hong Kong/2014	N		T	R		**S**		**S**		L		**N**		**Y**			K	**K**	**P**	D		**K**		E	**A**		P					
2017	*K*		*K*	R	*G*	S	*K*	–		–		–		–			*T*	–	*L*	–		–		–	–		–	77	89.5	100	100	100

A total of 32 amino acid substitutions were encountered in H3 proteins, of which 13 substitutions were located in HA head and 19 in the stem region (Table 7). The antigenic sites had the largest proportion of substitutions found in HA head domain with four substitutions in each A and B epitopes, two in C epitope and one in E epitope. Three substitutions were observed within the HA RBS: one in 190-helix (N189K) and two in 220-loop (V223I and N225D). All of which became dominant variants in viruses circulated in 2016.Two more substitutions were also observed close to RBS: the sporadically detected R142G/K substitution (A site) and fixed A198S substitution. Overall, 16 of detected substitutions were fixed in the trunk of the evolutionary tree and were found in all H3N2 viruses in 2017 (Fig. 4b). Three additional substitutions were also found in > 50% of H3N2 viruses in 2017. Herein, the amino acids were numbered using H3 numbering scheme exclusive of the signal peptide.

**Table 7:** Amino acid substitutions identified in the HA of H3N2 viruses sequenced during 2009–2017. Amino acid substitutions were identified in comparison to A/Brisbane/10/2007. Bordered amino acids indicate substitutions in receptor binding site. Deleterious mutations were predicted by online server PROVEAN and are indicated in bold. Only one HA sequence was deposited from MENA countries during 2010 hence this year was excluded from mutation and glycosylation analysis

Reference strain		AA substitution		Antigenic Site	2009 (%)	2011 (%)	2012 (%)	2013 (%)	2014 (%)	2015 (%)	2016 (%)	2017 (%)
Brisbane/2007	HA1	L3I						7	5	85	64	100
		Q33R					9	19	80	93	68	100
		S45 N				23	5	69	90	96	83	100
		T48I				23	5	54	87.5	91	72	100
		D53N		**C1**		9	32	30.7		4	4	20
		E50R							10			
		Q75H							10			
		N81K		**E2**			8				6	
		Y94H				9	38		12.5		4	
		N121K		**A1**						4	25.5	80
		T128A						31	72.5	6	12.7	
		R142	G	**A3**		9	2	30	72	10	12.7	30
			K				13.5				12.7	10
		N144	S	**A3**					5	87	40	60
			K								21	30
		N145S		**A3**		59	48.5	100	100	100	100	100
		L157S		**B1**	4	4		15	17.5	4	6	
		F159	Y	**B1**					15	87	62	100
			S						15		8	
		K160 T		**B1**						83	53	90
		N171K								6	19	60
		N189K		**B2**	73	100	100	100	100	100	100	100
		A198S				77	43	61.5	100	91	83	100
		T212A		**D**	63.6	95	100	84	100	91	85	100
		V223I				91	46	84	100	100	85	100
		N225D							20	89	68	100
		R261Q						15			8	20
		N278K		**C2**		9		54	97	93	74	100
		E280A				4	38				4	
		Q311H							7	85	59	60
		N312S			82	91	62	69	100	100	91	100
	HA2	V347	M			9		23	15			
			K			22			40	4		
		**G484E**								2	27.5	50
		D487N				59	43	23				
		D489N			9	13		15	12	87	64	100
Total number of substitutions/years					**5**	**17**	**16**	**19**	**23**	**25**	**28**	**23**

After 2009 pandemic, a few H3N2 viruses circulated carrying five amino acid substitutions compared to Brisbane/2007 vaccine strain. Most prevalent substitutions detected were N312S (82%) followed by N189K (73%) at B epitope and T212A (63.6%). Minority of viruses were also possessing L157S (4%) at B site and D489N (9%) substitutions. Unlike L157S, which was detected sporadically over years, D489N variant gradually replaced the parental variant in subsequent seasons before becoming the dominant variant in 2017(Fig. 4b). Only one HA sequence of H3N2 virus was deposited in IRD from MENA region during 2010 and was carrying N189K substitution. Despite the limited activity of H3N2 during 2011, various substitutions were introduced to H3N2 virus population. Besides previously reported N189K, T212A and N312S which became dominant variants (> 90%) in H3N2 viruses, 12 new substitutions were additionally identified during 2011. Among these, five substitutions were found at antigenic sites: D53N (9%) and N278K (9%) at C epitope; R142G (9%) and N145S (59%) at A epitope and L157S at B epitope. Further, six changes were introduced in 2011 and gradually predominated variants detected in later seasons: S45 N (23%), T48I (23%), A198S (77%) and V223I (91%) in HA1 subunit as well as D489N (13%) in HA2 subunit (Table 7). In 2012, only two novel substitutions were detected; N81K (8%) at E epitope and Q33R (9%). In contrast to N81K that was reported sporadically over years, the frequency of Q33R increased gradually in subsequent years before becoming characteristic of all H3N2 viruses in 2017. All viruses had the N189K and T212A, while approximately 50% of them were carrying N145S, A198S, V223I and D487N substitutions by end of 2012. Other sporadic substitutions were also detected such as R142G/K (15.5%) and D53N (32%) found in antigenic epitopes A and C, respectively (Table 7). The vaccine strain of the 2012–2013 season (Victoria/2011) was characterized by S45 N, T48I, N189K, A198S, T212A, V223I and N312S substitutions. Predominantly circulating viruses in 2013 carried these substitutions and additionally L3I (7%), T128A (31%) and R261Q (15%) substitutions. Unlike T128A and R261Q, L3I accumulated in H3N2 virus population and become a dominant variant in 2017. Of note, T128A mutation near A epitope of 17.6% of long circulated H3N2 viruses had been previously reported to be associated with high mortality rate among children [45]. Moreover, previously reported N278K and N145S were found in 54 and 100% of viruses, respectively. In 2014, majority of H3N2 viruses (> 75%) were already possessing 11 of previously identified substitutions (Fig. 4b). Circulated viruses were also characterized by the appearance of four novel substitutions; N144S (5%) F159Y/S (30%), N225D (20%) and Q311H (7%). All of these variants became fixed in the backbone of H3 phylogenetic tree with F159Y and N225D being found in all viruses isolated in 2017. Also, 10% of viruses emerged with E50R and Q75H substitutions were exclusively found in 2014. During 2015, circulating viruses were showing 97.2% similarity to respective vaccine strain (Texas/2012) (Table 7). Additional four novel substitutions were acquired: N121K (4%) at A epitope, K160 T (83%) at B epitope, N171K (6%) and G484E (2%). The frequencies of these substitutions increased dramatically in subsequent years reaching up to 80% for N121K and 90% for K160 T in 2017. In most cases, H3N2 viruses in 2015 possessed 17 fixed changes that have accumulated since 2009 (Fig. 4b). Towards 2016, viruses have become more stable carrying previously established substitutions and showing no novel mutation. In total, 28 amino acid substitutions were reported, however, 14 of them were already expressed in vaccine strain recommended for 2016 (Switzerland/2013). Likewise, no novel mutations were observed in HA protein during 2017. All H3N2 viruses were carrying 20 of fixed substitutions.

Overall, H3N2 viruses acquired fixed mutations rapidly. Since 2009, the number of fixed mutations increased dramatically from one fixed substitution in 2009 to 15 in 2017 (Fig. 4b). By the end of 2015, the prevalence of fixed mutations was > 80%. Interestingly, the fixed mutation, G484E, which appeared first in 2015 (2%) was predicted to exert a deleterious effect on HA function (provean score − 4.94). Sporadic substitutions were also introduced several times in the virus population since 2009, e.g. D53N, Y94H, T128A, L157S, R261Q and E280A in HA1 subunit and V347 M/K and D487N in HA2 subunit (Table 7). Other sporadic substitutions are listed in Table 7.

In addition to genetic drift, HA glycosylation pattern of H3N2 viruses has also changed since 2009. Nine putative N-glycosylation sites were found in all H3N2 isolates at 8, 22, 63, 126, 133, 165, 246, 285 and 483 (Fig. 5). However, the emergence of S45 N substitution in 2011 and K160 T substitution in 2015 was associated with acquisition of two additional glycosylation sequons at N45 (HA stem) and N158 (HA head) residues (Table 8). Glycosylation sequon at N45 and N158 were found in 23 and 5% of viruses in 2011 and 2015 respectively. However, number of viruses expressing these two sequons has increased gradually until found in all H3N2 viruses in 2017 (Table 8).

**Table 8:** Dynamics of HA and NA glycosylation for H3N2 viruses isolated from MENA region during 2009–2017. In total, glycosylation patterns of 239 HA and 132 NA sequences of H3N2 virus were analyzed. Numbers between brackets represent the percentage of sequences expressing glycosylation sequon in corresponding year while conserved glycosylation sites are indicated in bold

Localization of potential glycosylation sequons in HA and NA of H3N2 viruses												
HA												
		Amino acid position										
Year	Number of sequences analyzed	Stem					Head				Stem	
2009	22	**8**	**22**		**63**	**126**	**133**		**165**	**246**	**385**	**483**
2011	22	**8**	**22**	45(23%)	**63**	**126**	**133**		**165**	**246**	**385**	**483**
2012	37	**8**	**22**	45(5%)	**63**	**126**	**133**		**165**	**246**	**385**	**483**
2013	13	**8**	**22**	45(69%)	**63**	**126**	**133**		**165**	**246**	**385**	**483**
2014	40	**8**	**22**	45(92%)	**63**	**126**	**133**	158(5%)	**165**	**246**	**385**	**483**
2015	47	**8**	**22**	45(100%)	**63**	**126**	**133**	158(81%)	**165**	**246**	**385**	**483**
2016	47	**8**	**22**	45(80%)	**63**	**126**	**133**	158(53%)	**165**	**246**	**385**	**483**
2017	10	**8**	**22**	45(100%)	**63**	**126**	**133**	158(100%)	**165**	**246**	**385**	**483**
NA												
Year	Number of sequences analyzed	Amino acid position										
2009	5	**61**			**70**	**86**		**146**			**234**	
2011	22	**61**			**70**	**86**		**146**			**234**	367 (90.6%)
2012	32	**61**			**70**	**86**		**146**			**234**	367 (100%)
2013	8	**61**			**70**	**86**		**146**			**234**	367 (100%)
2014	33	**61**			**70**	**86**		**146**			**234**	367 (100%)
2015	23	**61**			**70**	**86**		**146**			**234**	367 (100%)
2016	8	**61**			**70**	**86**		**146**			**234**	367 (100%)

#### Molecular evolution of N2 protein

The NA gene was highly variable with total of 20 mutations detected in H3N2 strains included in this study compared to A/Brisbane/2007 virus (Additional file 4: Table S3). The catalytic and framework sites that support the catalytic residues within NA were highly conserved and are generally stable over time. However, eight substitutions were found near NA active site: Y155F, I176M, E221D, S245 N, S247 T, S367 N, K369 T and N402D. With the exception of Y155F and I176M, these substitutions were acquired by majority of H3N2 viruses (75%) in 2016. Importantly, none of the analyzed strains had the oseltamivir resistance sites H274Y, E119I or R292K [26]. Six potential glycosylation sites were predicted in the NA gene of 132 H3N2 strains, including N61, N70, N86, N146, N234 and N367. The appearance of two substitutions S367 N and K369 T has led to acquisition of a new glycosylation site at 367 position was found in all N2 sequences isolated after 2010 (Table 7).

#### Comparison of mutation accumulation at head and stem of HA protein in pH1N1 and H3N2 subtypes

**Fig. 6:**
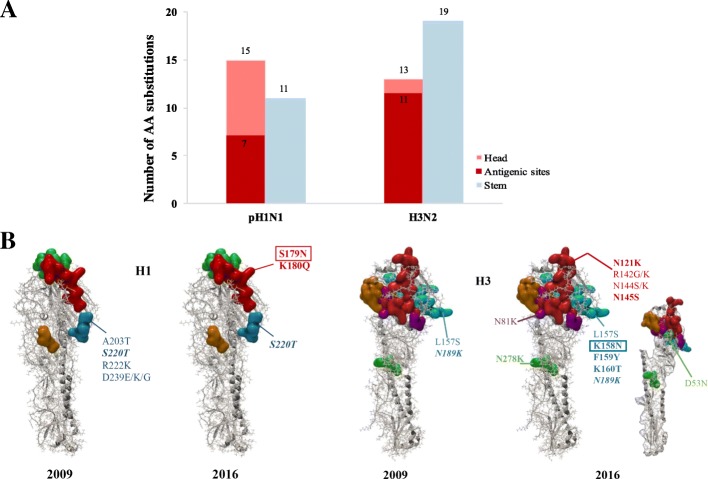
Amino acid (AA) substitutions in HAs of pH1N1 and H3N2 viruses circulated between 2009 and 2017. **a** Number of amino acid substitutions in HA head and stem domains of pH1N1 and H3N2 viruses. Amino acids are colored according to their location in head (red), stem (blue) and antigenic sites (dark red). **b** Amino acid substitutions identified in the antigenic sites of HAs of pH1N1 and H3N2 viruses in 2009 and 2016. The 3D crystal structures of A/California/04/2009 (PDB 3LZG) and for 2005 human H3N2 Virus (PDB 2YP7) are displayed using CLC genomic workbench version 11. The antigenic sites of H1 are colored: Sa (red), Sb (green), Ca (blue) and Cb (orange). The antigenic sites of H3 are colored: A (red), B (blue), C (green), D (orange) and E (purple). The inner site of H3 structure (2016) is shown to visualize D53N substitution in C1 epitope. Fixed substitutions developed during the evolution of the influenza viruses are labeled in bold. AA substitutions in RBS are shown in *italics*. AA substitutions that are associated with changes of potential glycosylation sites are framed

**Fig. 7:**
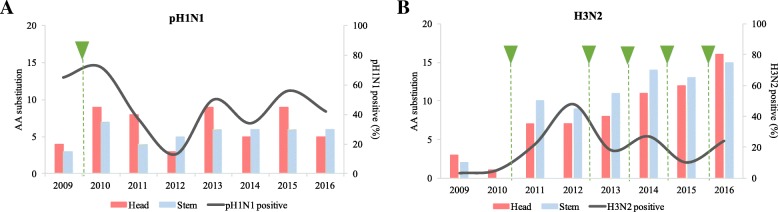
Amino acid substitutions in the HA genes of (**a**) pH1N1 and (**b**) A(H3N2) viruses circulated during 2009–2017. Amino acid substitutions in the head domain of the HA protein are labeled in red and amino acid (AA) substitutions in the stem domain are displayed in blue. The black curve shows the number of positive samples as reported to FluNet (WHO) during the same period. The green arrows denote the introduction of a new vaccine strain

When comparing the localization of substitutions in the HA protein of both subtypes, we found that most of substitutions in H3N2 were located in the stem (*n* = 19) whereas in pH1N1, we found the larger number of substitutions in the head region (*n* = 16) (Fig. 6a). Similarly, number of substitutions in antigenic site were much higher in H3N2 viruses (*n* = 12) compared to only 4 substitutions in pH1N1 viruses (Fig. 6a). Of the substitutions detected in antigenic sites during 2009, 3 were fixed in pH1N1 virus population compared to 7 fixed substitutions in H3N2 viruses in 2016 (Fig. 6b). Analysis of total number of fixed substitutions versus non-fixed substitutions in both subtypes revealed that the non-fixed substitutions predominated in pH1N1 (*n* = 18 out of 26) while fixed substitutions predominated in H3N2 (*n* = 20 out of 32). However, number of fixed substitutions in HA head and stem domains of both subtypes was equal. Interestingly, higher number of substitutions in HA head domain of pH1N1 virus was associated with higher number of pH1N1 positive cases during 2009–2011 and 2013–2016 (Fig. 7a). However, the higher number of substitutions in HA stem domain of pH1N1 was associated with a lower pH1N1 activity during 2012 and 2017. In contrast, the higher number of substitutions in HA stem was seen in H3N2 viruses during 2009–2016 was not associated with increased influenza activity (Fig. 7b).

## Discussion

We compared the relative contribution of influenza types (A and B) and subtypes (pH1N1 and H3N2) to influenza epidemics and investigated seasonality patterns in Arabian Gulf, Levant and North Africa regions during 2009–2017. Influenza surveillance data were retrieved from the global web-based tool ‘FluNet database’ which was launched first in 1997 by WHO for influenza surveillance [24]. A preliminary check of data available revealed that reported influenza cases were unevenly distributed across countries, in addition to lack of data from three countries (Saudi Arabia, Libya and Yemen), thus a total number of 17 countries were involved in current study. A study comparing influenza seasonality patterns among MENA countries has been published earlier in 2018 by Caini and his colleagues [46]. In the current study, we compared influenza seasonality based on WHO classification of influenza transmission zones and divided MENA countries in three main regions: Arabian Gulf (5 countries) and Levant (6 countries) which belong to Western Asia transmission zone and North Africa (5 countries) which is classified as North Africa transmission zone [33].

Influenza A was dominant in all years, with the pH1N1 accounting for the majority of cases except in 2012, during which H3N2 dominated. Influenza A and B accounted for 79.5 and 20.5% of all influenza cases during the study period, respectively. Generally, seasonality of influenza epidemics in all regions under study was in line with the northern hemisphere with a typical peak placed between December and March. Although Levant and Arabian Gulf lies in West Asia transmission zone (based on WHO classification), seasonality trends in Gulf were similar to that of North Africa rather than Levant. In all years, influenza activity started during October at high levels in Gulf and North Africa compared to a weak, late commence of the season in Levant, albeit the earlier winter seasons in the later. Furthermore, influenza positive cases were gradually increasing/decreasing in Gulf and North Africa, whereas a sharp increase/decrease of activity was reported in Levant (Additional file 2: Figure S1). The less defined seasonality pattern in Gulf countries could be partially explained by the tropical-like climate of these countries which is generally characterized by a wide primary peak in winter in addition to secondary smaller peaks seen throughout the year [34]. In contrast, the location of Levant countries (Turkey-Lebanon-Syria-Jordan-Cyprus) in temperate regions was demonstrated by a clear seasonal variations of influenza epidemics, with a marked peak in cold winter months [46–48].

In addition to monitoring activity of circulating influenza viruses, WHO utilizes surveillance data in studying virus evolution and assessing the antigenic characteristics of circulating viruses before selecting the most appropriate influenza vaccine strains for both hemispheres. Therefore, the molecular evolution of surface glycoproteins (HA and NA) of influenza A subtypes were also investigated in current study. All HA and NA sequences of pH1N1 and H3N2 viruses depicted in IRD since 2009 (total of 1260 sequences) were analyzed. Due to the limited number of sequences available, sequence analysis was done per MENA as whole rather than independent three regions. Phylogenetic analysis of both genes revealed that influenza viruses clustering in the phylogenetic tree is mainly chronological for both subtypes. Additionally, the phylogenetic analysis of the HA sequences revealed similar evolution of mutations circulating worldwide and hence similar clustering with globally circulating strains. In accordance to published data [25], majority of pH1N1 viruses after 2014 clustered within the genetic clade 6B and related sub-clade, 6B.1, while in H3N2 viruses’ sub-clades 3C.2 and related sub-clade 3C.2a were the predominating. In contrast to our findings, though, pH1N1 viruses belonging to group 6 related sub-clades (6A, 6B and 6C) were detected globally during 2013–2014 compared to an earlier appearance (2011–2012) in MENA countries. Moreover, group 6C pH1N1 viruses were not found after 2013 in MENA countries, however, were found in viruses isolated in other countries during 2015. Likewise, phylogenetic analysis of H3N2 viruses has revealed some differences compared to globally reported data [25]. Although global reports indicate that the majority of H3N2 viruses circulated during 2011–2012 fell into sub-clade 3C, viruses were mainly clustering within subclades 3A and 3B in MENA countries during that period.

For further evolution analysis of the pH1N1 and H3N2 HAs, we determined the positions of identified substitutions with respect to their localization in HA protein structure. Generally, more substitutions were detected in H3N2 viruses (*n* = 32) compared to pH1N1 viruses (*n* = 24) during study period. In accordance with our findings, analysis of antigenic dynamics of both subtypes’ HAs revealed that H3N2 viruses evolve faster than human H1 subtype [49]. The average amino acid mutation rate of the HA protein for H3N2 virus was 3.6 per year compared to 2.45 per year for H1N1 [50]. One reason could be the continuous circulation of H3N2 virus in human population since 1968, resulting in higher immune pressure as compared to the relatively new pH1N1 virus [51]. Substitutions were most frequently found in the head domain in pH1N1 viruses, including eight changes in the antigenic sites and five in the RBS, while interestingly, changes were mainly localized in stem domain in H3N2 viruses. Yet, most of substitutions identified in head domain of H3N2 viruses were localized in or near major antigenic sites (*n* = 8). Of note, changes of the antigenic HA composition of H3N2 viruses were accompanied by a six-time exchange of the H3N2 vaccine strains which exert more pressure on H3 antigenic sites compared to only two changes in pH1N1 vaccine strains during study period (Additional file 1: Table S1). Overall, the H3N2 strains displayed more diversity and faster accumulation of mutations in antigenic epitopes compared to pH1N1 strains [49]. HA sequences from the H3N2 strains possessed two amino acid changes in one epitope (B; L157S and N189K) during the 2009. In 2011, this number increased to seven changes in three epitopes: two in epitope A, three in epitope B, and two in epitope C (Fig. 4b). In following years, more antigenic drift occurred due to changes in H3 antigenic site, however, most of these changes were found in recommended vaccine strain of subsequent seasons. The continuous change in H3N2 vaccine strains could also explain H3N2 lower activity despite the larger number of accumulated substitutions (Fig. 7b). Reports measuring influenza vaccine effectiveness have shown that the overall vaccine effectiveness (VE) was highest during 2010–2011 season (60%), while lowest during the 2014–2015 season (19%) [52]. This was associated with change in vaccine strain of H3N2 from A/Victoria/361/2011 to A/Texas/50/2012. Thus, vaccine composition was reformulated again for 2015–2016 season to contain A/Switzerland/2013 and was shown to be more effective (48%) [52]. In 2015, H3N2 viruses were carrying four substitutions in antigenic sites that were not found in A/Texas/2012 strain including: N144S and N145S in A3 site, and Y159F and K160 T in B1 site. Moreover, 87% of pH1N1 viruses had acquired S179 N mutation in 2015, which was associated with acquisition of potential glycosylation site. Together, these changes in H3N2 and pH1N1 viruses isolated in 2015 might -at least- partially explain the lower vaccine effectiveness during earlier months of 2015.

The pandemic strain, pH1N1, originated from swine reservoir and circulated in human population for much shorter time (~ 9 years) compared to H3N2 viruses that has been circulating since 1968. Therefore, pH1N1 viruses are still considered to be in adaptive phase in terms of recognition of new receptor as well as avoiding immune response of the new host. Evidence of such adaptation process was revealed while analyzing mutations acquired by different pH1N1 viruses since 2009. Positive selection test of HA gene in pH1N1 viruses revealed that two amino acids in Ca antigenic site (D239G/E/N and Q240R) showed evidence of adaptive evolution. Owing to its presence in HA antigenic and RBS, variants of 239 position, in particular, has been found to be a key determinant of virus capability to bind epithelial cells in upper and/or lower respiratory tract [53]. D239G variant has demonstrated the ability to bind α2–6 receptors in addition to increased binding affinity for α2–3 receptors expressed by pneumocytes in lower respiratory tract, and hence resulting in enhanced illness [37]. 225G and 225 N variants were only detected in specimens obtained from patients with severe clinical symptoms, whereas D225E variants were detected in both severe and mild cases with similar frequencies [53, 54]. In this study, D239 variants occurred in 10.3% of pH1N1 viruses. In contrast to D239E (8%) which appeared during 2009–2010, D239G and D239N substitutions formed no genetic cluster and reported sporadically suggesting that there was no spreading of viruses carrying this variant. Of note, variants of D239 position were also reported in seasonal strains circulated before 2009 [39]. Receptor binding site of HA protein represented by 130-loop (148–152), 190-helix (201–208) and 220-loop (235–242) (H1 numbering) is one of the major determinants of virus transmission ability [55]. Therefore, amino acid residues in and around the receptor binding site were predicted to change during adaptation process to human receptors. Those amino acids include: S200, S202, D204, A214, I233 and E241 [56]. Four of these sites were found to be mutated in pH1N1 viruses included in this study. Of these, S200P, S202 and A214T were detected sporadically while I233T was introduced successfully in more than 80% of viruses after 2014. In 2013, de Vries and his colleagues has reported that S200P and S202 T substitutions would increase the receptor-binding avidity whereas the presence of A214T substitution was linked to decrease binding avidity [57]. Interestingly, sequences expressing A214T variant were always associated with S200P and S202 T, which could be considered as compensatory process. With respect to I233T mutation, the presence of non-polar Isoleucine in 233 position has been found to disrupt the positioning of residues in the RBS from making a stable network of interactions [58]. The substitution the Isoleucine with the polar amino acid Lysine, has been shown to significantly increase HA affinity to human receptor [58]. Similarly, the displacement of the non-polar Isoleucine residue at 233 position with a polar amino acid, Threonine, could generate a stable ionic interaction between polar T233 residue in RBS and the acidic E241 residue in 220 loop, consequently increasing binding affinity to human receptor in comparison to earlier pH1N1 isolates [58]. In addition to mutations in RBS, limited number of mutations in HA stem domain were also acquired in an early stage of pH1N1 evolution and thereafter introduced successfully in virus population. Among these mutations are E391K and E516K, which are found in the vicinity of the fusion peptide and transmembrane domain respectively. Such mutations are usually considered to be under negative selection because they might alter HA stability or functions [59]. However, for a newly emerged virus such as pH1N1, such mutations may be prone to positive selection because it has to adapt to the new host. It is noteworthy, both subtype viruses acquired fixed mutations generally rapidly, however, more fixed mutations were accumulated in H3N2 compared to more non-fixed substitution in pH1N1, another evidence that the latter is still in adaptation process.

In addition to antigenic drift, glycans on the globular head of HA can shield antigenic sites from neutralizing antibodies, but at the same time can affect HA binding to host cell receptors [38]. Compared to vaccine strains, two amino acid mutations involving S179 N (Sa epitope) and K160 T (B epitope) in globular head of H1 and H3, respectively, have resulted in acquisition of putative glycosylation sequons. Glycosylation at the antigenic sites of HA head could be associated with increased viral pathogenicity by hiding antigenic sites from immune recognition. Interestingly, S179 N glycosylation was also reported in seasonal H1N1 viruses circulated during 1940–1948 before being replaced by N177 glycosylation in subsequent seasons until 2009 [60]. Additional glycosylation sequons in positions 42 and 367 have been also acquired by N1 and N2 respectively. Unexpectedly, mutation rate of N2 (3.09 × 10^− 3^) was found to be higher than that of H3 (1.47 × 10^− 3^), however, this could be related to unusual number of mutations in N2 sequences which were not reported elsewhere. That in addition to low number of N2 sequences which were mainly deposited from single country (Iran).

Systematic monitoring for influenza viruses is also important for evaluating the efficacy of the influenza NA inhibitors. Currently, neuraminidase inhibitors (NAIs) such as oseltamivir and zanamivir are the main drugs for treatment of influenza infections [61]. The effectiveness of oseltamivir in treating influenza infections was threatened by the predominance of oseltamivir resistance among seasonal H1N1 viruses 2009–2010, even in countries where oseltamivir had not been used [62]. However, seasonal virus was subsequently displaced by the pH1N1 virus, which is largely sensitive to oseltamivir [63]. It is noteworthy, oseltamivir-resistant strains were rarely reported during 2009–2010 [64]. In following years, though, a notable increase in the proportion of oseltamivir-resistant pH1N1 viruses were reported worldwide amongst patients with or without NAI treatment [65, 66]. In accordance with our findings, most of reported oseltamivir-resistant cases exhibited geographical and temporal clustering. Such cases were reported during 2011 in New England, Australia [67] and during 2013 in Sapporo, Japan [68]. Here, all oseltamivir-resistant strains were reported were clustering in Oman and Iran during 2013 and 2015, respectively, suggesting efficient transmission of oseltamivir-resistant viruses among humans [69]. This raises the concern that prevalence of oseltamivir-resistant pH1N1 viruses may increase in the future as the case of the previously circulating seasonal H1N1 viruses. Interestingly, two of NA fixed mutations reported in current study, V241I and N369K, have been found to offer robust fitness on pH1N1 viruses carrying H275Y mutation by enhancing NA surface expression and its enzymatic activity [44]. The presence of such permissive mutations in all pH1N1 viruses suggesting that they are more permissive to the acquisition of H275Y and hence increase the risk of oseltamivir-resistant strains to spread globally. Here, we found oseltamivir-resistance mutation, H275Y, in only 4% of pH1N1 virus while no resistance mutations were reported in H3N2 viruses besides both subtypes have retained susceptibility to zanamivir. Unfortunately, we were unable to find published resources or reports regarding the prevalence of NAIs use in MENA countries.

There are limitations in this study. Surveillance data was not available from Saudi Arabia, Yemen or Libya. The lack of influenza surveillance data from Saudi Arabia especially during Umra and Haj seasons could underestimate the actual prevalence of influenza infection in MENA region. Nevertheless, several studies investigating the burden of respiratory infection during Hajj season were published [70]. Moreover, there is a relatively small number of HA and NA genes depicted in Influenza Research Database form MENA countries, especially for H3N2 viruses. This mandates further molecular characterization of circulating viruses in the region. Analysis of small number of sequences could preclude exploring the actual genetic diversity of influenza viruses.

## Conclusion

In the context of the national influenza surveillance, we estimated the epidemiology of influenza types A and B in Arabian Gulf, Levant and North Africa regions since 2009. The pandemic strain pH1N1 was the dominant subtype in all years except in 2012 which was dominated by H3N2. Although WHO includes both Gulf and Levant in Western Asia, we found that seasonality patterns of Gulf and North Africa regions are similar compared to Levant region. We have also characterized the genetic evolution of influenza A subtypes: pH1N1 and H3N2 viruses during same period. Comparison of identified mutations of pH1N1 and H3N2 viruses encountered over 9 years revealed significant differences with regard to position and function of identified substitutions. pH1N1 viruses accumulated more substitutions in the head domain whereas H3N2 viruses had most of the reported substitutions in the stem domain. Moreover, H3N2 viruses acquired more fixed substitutions that were successfully introduced in viral population compared to larger number of non-fixed substitutions in pH1N1. More importantly, amino acids in the antigenic sites of H3N2 viruses were showing more variability than those of pH1N1 viruses, probably indicating greater immunological pressure. Interestingly, the increase of substitutions in HA globular head of pH1N1 virus was seen in strong influenza activity while that was not the case in H3N2 viruses despite the higher number of accumulated mutations. A possible explanation could be that the H3N2 viruses have been circulating in human population since 1968 resulting in circulation of different variants of virus compared to the relatively novel pH1N1. On the other hand, molecular analysis of the neuraminidase genes revealed the clustering of Y275H oseltamivir resistant mutation in pH1N1 viruses isolated from Oman and Iran, however, neither NAs carried any of substitutions associated with reduced susceptibility to zanamivir. Major challenges are yet to be faced by MENA countries in order to contribute to global surveillance of influenza virus by improving their surveillance systems for detection and response to all public health events.

## Additional files

Additional file 1: **Table S1.** Accession numbers of pH1N1 and H3N2 vaccine and representative strains as obtained from NCBI and GISAID. Vaccine strains were used as references when analyzing HA and NA sequences. Representative stains were used to identify the clades and sub-clades in phylogenetic tree. (DOCX 31 kb)

Additional file 2: **Figure S1.** Seasonality patterns of influenza viruses in MENA during 2009–2017. Here, we are presenting influenza activity from September to April of each year in Arabian Gulf, Levant and North Africa regions. Monthly trend of each subtype is presented by bars: red bars represents percentage of pH1N1 positive cases, Blue represents H3N2 positive cases and green represents Flu B positive cases. (DOCX 159 kb)

Additional file 3: **Table S2.** Accumulation of amino acid substitutions in N1 protein of pH1N1 viruses of during 2009–2017: Amino acid substitutions were identified relative to A/California/07/2009 vaccine strain. The last column shows the overall prevalence of each substitution throughout study period (2009–2017). Substitutions associated with NAIs activity are indicated in bold. N1 numbering was used for reporting substitutions. (DOCX 34 kb)

Additional file 4: **Table S3.** Accumulation of amino acid substitutions in NA protein of in N2 protein of H3N2 viruses of during 2009–2017: Amino acid substitutions were identified relative to A/Brisbane/2007 vaccine strain. The last column shows the overall prevalence of each substitution in all NA sequences included in study (2009–2017). N2 numbering was used for reporting substitutions. (DOCX 34 kb)

## Abbreviations

HA: Hemagglutinin
IRD: Influenza Research Database
MENA: Middle East and North Africa
NA: Neuraminidase
NAIs: Neuraminidase inhibitors
RBS: Receptor Binding Site
