# Supplementary material for: Epidemiological and genetic characterization of pH1N1 and H3N2 influenza viruses circulated in MENA region during 2009–2017
Source: BMC Infect Dis. 2019 Apr 11;19:314. doi: 10.1186/s12879-019-3930-6 (PMC6458790; doi:10.1186/s12879-019-3930-6)
Supplement: Supplementary file 1 — Table S1. Accession numbers of pH1N1 and H3N2 vaccine and representative strains as obtained from NCBI and GISAID. Vaccine strains were used as references when analyzing HA and NA sequences. Representative stains were used to identify the clades and sub-clades in phylogenetic tree. (DOCX 31 kb) [file 12879_2019_3930_MOESM1_ESM.docx]

**Additional file 1**

**Table S1: Accession numbers of pH1N1 and H3N2 vaccine and representative strains used for phylogenetic analysis as obtained from NCBI and GISAID.** Vaccine strains were used as references when analyzing HA and NA sequences. Representative stains were used to identify the clades in phylogenetic tree. All representative strains were used as suggested by Worldwide influenza center of Francis Crick Institute.

| **pH1N1** | | | **H3N2** | | |
| --- | --- | --- | --- | --- | --- |
| **Vaccine strain** | **HA** | **NA** | **Vaccine strain** | **HA** | **NA** |
| A/California/07/2009 | KF009554 | KC781784 | A/Brisbane/10/2007 | KM978061 | KM978075 |
|  |  |  | A/Perth/16/2009 | KJ609206 | CY081429 |
|  |  |  | A/Victoria/361/2011 | KC306165 | KJ942682 |
|  |  |  | A/Texas/50/2012 | KC892952 | KC892281 |
|  |  |  | A/Switzerland/2013 | EPI530687 | EPI530688 |
| A/Michigan/45/2015 | KY117023.1 | KY117025.1 | A/Hong Kong/4801/2014 | EPI1539576 | EPI614436 |
| **Representative reference strains used in phylogeny** | | | | | |
| EPI319447 | EPI326206 | EPI278607 | EPI326139 | EPI358885 | EPI346607 |
| EPI319590 | EPI320141 | EPI382424 | EPI426061 | EPI467994 | EPI1460558 |
| EPI319527 | EPI464862 | EPI498431 | EPI574644 | EPI426077 | EPI335923 |
| EPI697729 |  |  | EPI405940 | EPI769531 | EPI530677 |
