# Supplementary material for: Epidemiological and genetic characterization of pH1N1 and H3N2 influenza viruses circulated in MENA region during 2009–2017
Source: BMC Infect Dis. 2019 Apr 11;19:314. doi: 10.1186/s12879-019-3930-6 (PMC6458790; doi:10.1186/s12879-019-3930-6)
Supplement: Supplementary file 2 — Figure S1. Seasonality patterns of influenza viruses in MENA during 2009–2017. Here, we are presenting influenza activity from September to April of each year in Arabian Gulf, Levant and North Africa regions. Monthly trend of each subtype is presented by bars: red bars represents percentage of pH1N1 positive cases, Blue represents H3N2 positive cases and green represents Flu B positive cases. (DOCX 159 kb) [file 12879_2019_3930_MOESM2_ESM.docx]

**Additional file 2**

**Figure S1: Monthly trends and seasonality patterns of influenza viruses in MENA during 2009-2017.** The graphs demonstrate influenza activity from September to April of each year in Arabian Gulf, Levant and North Africa regions. Red color represents percentage of pH1N1 positive cases, Blue represents H3N2 positive cases and green represents Flu B positive cases.
