# Supplementary material for: Epidemiological and genetic characterization of pH1N1 and H3N2 influenza viruses circulated in MENA region during 2009–2017
Source: BMC Infect Dis. 2019 Apr 11;19:314. doi: 10.1186/s12879-019-3930-6 (PMC6458790; doi:10.1186/s12879-019-3930-6)
Supplement: Supplementary file 3 — Table S2. Accumulation of amino acid substitutions in N1 protein of pH1N1 viruses of during 2009–2017: Amino acid substitutions were identified relative to A/California/07/2009 vaccine strain. The last column shows the overall prevalence of each substitution throughout study period (2009–2017). Substitutions associated with NAIs activity are indicated in bold. N1 numbering was used for reporting substitutions. (DOCX 34 kb) [file 12879_2019_3930_MOESM3_ESM.docx]

**Additional file 3**

**Table S2: Amino acid substitutions identified in NA of pH1N1 during 2009-2017:** Amino acid substitutions were identified relative to A/California/2009 vaccine strain. The last column denotes the overall prevalence of each substitution throughout study period (2009-2017). Substitutions associated with NAI activity are indicated in **bold**. N1 numbering was used for reporting substitutions.

| **N1** | | | | | | | | | | | |
| --- | --- | --- | --- | --- | --- | --- | --- | --- | --- | --- | --- |
| **Reference strain** |  | **AA substitution** | **2009** (%) | **2010** (%) | **2011** (%) | **2012** (%) | **2013** (%) | **2014** (%) | **2015** (%) | **2016** (%) | **Substitution Frequency (2009-2016) (%)** |
| **California/2009** | **Antigenic site** | I46T | 11 |  |  |  |  |  |  |  | 3 |
|  |  | N68T | 18 |  |  |  |  |  |  |  | 5 |
|  |  | K102Q |  |  |  |  |  |  | 6 |  | 1.4 |
|  |  | V106I | 100 | 100 | 100 | 45 | 25 | 4 | 7 |  | 57.7 |
|  |  | I117M |  |  |  |  |  | 20 | 7.5 |  | 3 |
|  |  | I188T |  | 2 |  |  |  | 8 | 1 |  | 1 |
|  |  | N270K |  | 2 |  |  |  | 44 | 51 |  | 10 |
|  |  | **H275Y** | 1 |  |  | 3 | 15.6 |  | 10 |  | 4 |
|  |  | N341D |  |  |  |  | 3 |  | 13 |  | 2.6 |
|  |  | K432E |  |  |  |  |  | 88 | 74 | 100 | 23.6 |
|  |  | E462K | 20 |  |  |  |  |  |  |  | 5.8 |
|  |  | G454S | 2 |  |  | 10 |  |  |  |  | 1 |
|  |  | D451G |  |  | 5 | 90 |  | 12 | 1 |  | 5 |
|  |  | T452I |  |  |  |  | 3 |  | 5 |  | 1 |
|  |  | N200S |  |  |  | 55 | 72 | 88 | 72 | 100 | 34 |
|  |  | N248D | 97 | 100 | 100 | 100 | 90 | 92 | 83 |  | 93 |
|  |  | V241I | 1 | 50 | 83 | 100 | 90.6 | 99 | 94 |  | 60 |
|  |  | I314M |  |  |  |  |  |  | 60 |  | 14 |
|  |  | N369K |  | 48 | 83 | 100 | 90 | 96 | 74 | 100 | 47 |
|  |  | N386K |  |  |  |  |  | 68 | 74 |  | 22 |
