# Supplementary material for: Epidemiological and genetic characterization of pH1N1 and H3N2 influenza viruses circulated in MENA region during 2009–2017
Source: BMC Infect Dis. 2019 Apr 11;19:314. doi: 10.1186/s12879-019-3930-6 (PMC6458790; doi:10.1186/s12879-019-3930-6)
Supplement: Supplementary file 4 — Table S3. Accumulation of amino acid substitutions in NA protein of in N2 protein of H3N2 viruses of during 2009–2017: Amino acid substitutions were identified relative to A/Brisbane/2007 vaccine strain. The last column shows the overall prevalence of each substitution in all NA sequences included in study (2009–2017). N2 numbering was used for reporting substitutions. (DOCX 34 kb) [file 12879_2019_3930_MOESM4_ESM.docx]

**Additional file 4**

**Table S3: Amino acid substitutions identified in NA of H3N2:** Amino acid substitutions were identified relative to A/Brisbane/2007 vaccine strain. The last column denotes the overall frequency of each substitution in all NA isolated during 2009-2017. N2 numbering was used for reporting substitutions.

| **Reference strain** | **AA substitution** | **2009** (%) | **2010** (%) | **2011** (%) | **2012** (%) | **2013** (%) | **2014** (%) | **2015** (%) | **2016** (%) | **Substitution Frequency (2009-2016)**  **(%)** |
| --- | --- | --- | --- | --- | --- | --- | --- | --- | --- | --- |
| **Brisbane/2007** | Y40C |  |  | 4 | 12 |  |  |  |  | 9 |
|  | L81P |  |  | 77 | 56 | 100 | 100 | 100 | 100 | 81 |
|  | D93G |  |  | 18 | 6 | 62 | 100 | 100 | 100 | 75 |
|  | V143M |  |  | 4 | 3 |  |  | 8 | 37 | 5 |
|  | Y155F |  |  |  |  | 25 | 69 |  |  | 18 |
|  | I176M |  |  | 4 | 44 |  |  |  |  | 11 |
|  | R210K |  |  |  | 47 |  |  |  |  | 10 |
|  | E221D |  |  | 4 |  | 37 | 30 | 100 | 100 | 34 |
|  | S245N |  |  |  |  |  |  | 49 | 75 | 14 |
|  | S247T |  |  |  |  |  |  | 49 | 87 | 14 |
|  | D251V |  |  |  |  | 25 | 63 |  |  | 17 |
|  | T267K |  |  |  |  |  | 6 | 87 | 75 | 21 |
|  | S315G |  |  |  |  | 25 | 60 |  |  | 17 |
|  | D339N |  |  |  |  |  |  | 4 | 62 | 4 |
|  | S367N |  |  | 100 | 100 | 100 | 100 | 100 | 100 | 96 |
|  | K369T |  |  | 100 | 100 | 100 | 100 | 100 | 100 | 96 |
|  | I380V |  |  |  |  |  | 3 | 91 | 50 | 20 |
|  | I239T |  |  | 4 |  | 12 | 18 | 13 | 12 | 9 |
|  | N402D |  |  | 82 | 87 | 75 | 100 | 100 | 100 | 88 |
|  | G414S |  |  |  | 15 |  |  |  |  | 4 |
|  | I464L |  |  | 96 | 53 | 62 | 100 | 100 | 100 | 81 |
